# 30 Years of Experience in the Management of Stage III and IV Epithelial Ovarian Cancer: Impact of Surgical Strategies on Survival

**DOI:** 10.3390/cancers12030768

**Published:** 2020-03-24

**Authors:** Berenice Delga, Jean-Marc Classe, Gilles Houvenaeghel, Guillaume Blache, Laura Sabiani, Houssein El Hajj, Nicole Andrieux, Eric Lambaudie

**Affiliations:** 1 Institut Paoli Calmettes, Department of Surgical Oncology, 13009 Marseille, France; berenice.delga@gmail.com (B.D.); houvenaeghelg@ipc.unicancer.fr (G.H.); blacheg@ipc.unicancer.fr (G.B.); sabianil@ipc.unicancer.fr (L.S.); elhajjh@ipc.unicancer.fr (H.E.H.); 2 Institut René Gauducheau, Site Hospitalier Nord, 44800 St Herblain, France; jean-marc.classe@ico.unicancer.fr (J.-M.C.); nicole.andrieux@ico.unicancer.fr (N.A.); 3 Faculty of Medical Sciences, Aix-Marseille University, CNRS, Inserm, CRCM, 13005 Marseille, France

**Keywords:** ovarian cancer, surgery, survival, prognosis

## Abstract

*Objective*: to analyze the evolution of surgical techniques and strategies, and to determine their influence on the survival of patients with stage III or IV epithelial ovarian cancer (EOC). *Methods*: a retrospective data analysis was performed in two French tertiary cancer institutes. The analysis included clinical information, cytoreductive outcome (complete, optimal and suboptimal), definitive pathology, Overall Survival (OS), and Progression-Free Survival (PFS). Three surgical strategies were compared: Primary Cytoreductive Surgery (PCS), Interval Cytoreductive Surgery (ICS) after three cycles of Neo-Adjuvant Chemotherapy (NAC), and Final Cytoreductive Surgery (FCS) after at least six cycles of NAC. We analyzed four distinct time intervals: prior to 2000, between 2000 and 2004, between 2005 and 2009, and after 2009. *Results*: data from 1474 patients managed for International Federation of Gynecology and Obstetrics (FIGO) stages III (80%) or IV (20%) EOC were analyzed. Throughout the four time intervals, the rate of patients who were treated only medically increased significantly (10.1% vs. 22.6% *p* < 0.001). NAC treatment increased from 20.1% to 52.2% (*p* < 0.001). Complete resection rate increased from 37% to 66.2% (*p* < 0.001). Of our study population, 1260 patients (85.5%) underwent surgery. OS was longer in cases of complete cytoreduction (Hazard Ratio (HR) = 2.123 CI 95% [1.816–2.481] *p* < 0.001) but the surgical strategy itself did not affect median OS. OS was 44.9 months, 50.3 months, and 42 months for PCS, ICS, and FCS, respectively (*p* = 0.410). After adjusting for surgical strategies (PCS, ICS, and FCS), all patients with complete cytoreduction presented similar OS with no significant difference. However, PFS was three months shorter when FCS was compared to PCS (*p* < 0.001). Conclusion: In our 30 years’ experience of EOC management, complete resection rate was the only independent factor that significantly improved OS and PFS, regardless of the surgical strategy.

## 1. Introduction

Epithelial Ovarian cancer (EOC) is the leading cause of death among gynecological cancers and the fifth leading cause of death among cancers in developed countries [1,2,3,4]. This can be attributed to the lack of early presenting symptoms: 60% of patients are diagnosed at advanced stages (International Federation of Gynecology and Obstetrics, FIGO stages III or IV) [4,5]. The five year survival rate in the USA in 2013 was 46.5% for all stages combined, 28.9% and 25% for stages III and IV, respectively [2,3,5].

Taxanes and platinum-based chemotherapy associated with aggressive surgery are the cornerstones of the management of EOC [6,7,8,9,10,11]. The absence of residual disease after cytoreductive surgery remains a major prognostic factor. The timing and the completeness of surgery impact the prognosis and the selection of the systemic treatment. The therapeutic arsenal was expanded with addition of Bevacizumab in adjuvant treatment and the addition of Poly ADP-ribose polymerase (PARP) inhibitors in maintenance therapy for patients with BRCA1/2 mutations [12,13,14,15].

Trials performed by Vergote et al. [16,17] and Kehoe et al. [18] showed no significant difference in survival rates between patients operated for Primary Cytoreductive Surgery “PCS” (prior to adjuvant chemotherapy) and Interval Cytoreductive surgery “ICS” (surgery after three or four cycles of neo-adjuvant chemotherapy (NAC)). Moreover, recent results of a randomized study showed beneficial outcomes when hyperthermic intraperitoneal chemotherapy (HIPEC) was associated with ICS [19]. When complete cytoreduction is deemed not possible during PCS or ICS, surgery can be performed after six cycles of chemotherapy as a Final Cytoreductive Surgery (FCS). This strategy has not been well evaluated in literature [20,21]. Since the poor survival rate for EOC is largely due to the lack of evidence concerning the best surgical timing and strategy, we conducted this retrospective bi-centric observational study.

The main objectives of this study are the assessment of the evolution of practices and the influence of the surgical strategy on survival outcomes for patients with advanced EOC (FIGO Stages III and IV).

## 2. Patients and Methods

### 2.1. Population Description

We conducted a retrospective bi-centric study at the Nantes and Marseille regional tertiary cancer centers in France between 1985 and 2015. The study protocol was approved by both institutions’ review boards (ICO and IPC Comité d’Organisation Stratégique) and was conforming to the French ethical standards and the 2008 Helsinki declaration.

Eligible patients were those with a confirmed diagnosis of a stage III or stage IV EOC. For the Nantes cancer institute, data was included from 1985 to 2015, and for the Marseille cancer institute, data was collected from 1993 to 2015. All patients underwent either surgery or chemotherapy or a combination of both. Patients were excluded if they had a non-epithelial ovarian cancer, a borderline, or a benign tumor. Patients who underwent a diagnostic surgery were not included in the study. Patients deemed inoperable and treated with only chemotherapy were not excluded from the study.

All patients underwent surgery with the intent to achieve complete cytoreduction. Surgical procedures included hysterectomy with bilateral salpingo-oophorectomy, omentectomy, para-aortic, and pelvic lymph-node dissection (patients included in the CARACO trial—NCT01218490—were randomized for lymph node dissection or no lymph node dissection) [22]. Appendectomy was performed in case of mucinous tumors. Complete resection of peritoneal carcinomatosis was performed even if bowel resection was required.

Treatment strategies were divided into four categories: (1) PCS followed by at least six cycles of platinum based adjuvant chemotherapy, (2) ICS performed after three to four cycles of NAC, and followed by at least two to three cycles of platinum-based adjuvant chemotherapy, (3) FCS performed after at least six cycles of NAC, followed (or not) by adjuvant chemotherapy, and (4) chemotherapy alone (inoperable patients).

In order to analyze the evolution of EOC management, four periods of time were defined:

P1 is the period of time before 2000, during which taxanes molecules were progressively introduced in EOC management.

P2 is the period of time between 2000 and 2004, during which we witnessed the systematic association of taxanes and optimal surgery.

P3 is the period of time between 2005 and 2009, during which we witnessed a progressive evolution towards complete cytoreduction.

P4 is the period of time after 2009, which is concomitant with the endorsement of complete cytoreduction surgery (CC0) in our practices and the progressive adoption of NAC and ICS.

According to the guidelines, post therapeutic follow-up included physical examination associated with CA125 serum level measurements every three months for two years, then every six months for the next three years, and annually thereafter.

### 2.2. Clinical and Pathological Features

Clinical and pathological data were prospectively collected in both cancer centers. The following characteristics were collected for each patient: age at diagnosis, date of first clinical visit, and characteristics at diagnosis (CA125, histological type, initial cytology, Silverberg grade or histologic grade, and FIGO stage), characteristics of surgery (date of surgery, procedures performed, surgical strategy (PCS, ICS or FCS), residual disease at the end of surgery), and characteristics of the systemic treatment (Neoadjuvant or adjuvant chemotherapy, number of cycles, protocols, and inclusion in clinical trials).

Overall survival (OS) was defined as the duration in months from surgery to death from any cause. Progression free survival (PFS) was defined as the duration in months from the surgical date to any recurrence of the cancer or death from any cause.

### 2.3. Statistical Analysis

Follow-up time was measured as the time elapsed between the date of diagnosis and the date of last-follow-up. The impact on survival of the above-cited factors was assessed by univariate analysis. The p-values were estimated using the Wald test. After univariate analysis, we performed a multivariate analysis using a logistical regression for factors influencing the surgical strategy and their impact. A COX model was applied to study factors that have an independent effect on survival. Data concerning patients without disease progression or death at last follow-up were censored. Survival curves were estimated using the Kaplan–Meier method, and compared with the log–rank test. The Pearson’s Chi-squared (Chi 2) test (categorical variables) and Wilcoxon test (continuous variables) were used to compare descriptive items. All statistical tests were two-sided with a 5% level of significance and performed with SPSS 24.0 (Statistical Package for Social Sciences; SPSS, Inc, Chicago, IL, USA).

## 3. Results

### 3.1. Clinical and Pathological Features

Between 1985 and 2015, 1474 patients treated for EOC with FIGO stages III (79.4%) or IV (20.6%) were included in the study. Median age at diagnosis was 61 years old (Range: 17–94). Surgical resection was performed for 1260 patients (85.5%): 52.3% underwent PCS, 14.1% ICS, and 33.6% FCS (Appendix A) = Flowchart.

Among the 1260 patients who underwent surgery, complete cytoreduction was achieved in 815 (64.7%), and 353 (28%) presented residual disease at the end of surgery. The rate of complete cytoreduction was significantly influenced by the surgical strategy (*p* < 0.0001) (Table 1b). Complete cytoreduction was achieved in 62.1% of PCS cases, 81.7% of ICS cases, and 78.1% for FCS cases. Information about residual disease after surgery was missing in 92 cases (7.3%) and these cases were excluded from the present analysis.

Moreover, 1076 patients received a platinum and taxane based chemotherapy, out of which 91% received carboplatin and paclitaxel (Table 1a). In 84% of the cases, NAC regimens were based on a combination of carboplatin and paclitaxel. Other chemotherapy regimens consisted mostly of a combination of platinum and cyclophosphamide that was administered to 227 of the patients before 2000. Other molecules used consisted of 5FU, Adriamycin, irinotecan. Data was missing for 35 patients, and these patients were excluded from the analysis.

### 3.2. Survival

Of the 1474 patients included in the study, median OS was 39 months [IC95%: 36–42], two years OS rate was 65.5%, five years OS rate was 34.3% and median PFS was 10.5 months.

1. In univariate analysis, cytoreductive surgery and the FIGO stage at diagnosis had a significant impact on OS: patients who underwent surgery presented a median OS of 44.4 months versus 14.7 months for those who did not (*p* < 0.001), patients diagnosed with stage III EOC presented a 42.7 months median OS versus 24.5 months for patients with stage IV (*p* < 0.001).

The five-year survival rate was 40% for FIGO stage III EOC and 29% for stage IV patients. Age was a significant prognostic factor, since the five-year OS rate was 46.6% for patients less than 50 years old, 37.1% for patients 50–74 years old, and 26.4% for patients above 75 (*p* < 0.0001).

2. Concerning the 1260 patients (85.5%) who underwent surgery, median OS was respectively 55.1 months for patients with complete cytoreduction versus 24.6 for patients with residual disease at the end of surgery (*p* < 0.001). Median OS for PCS was 44.9 months versus 50.3 months for ICS and 42 months for FCS (*p* = 0.410).

3. Among patients with complete cytoreductive surgery, the surgical strategy was not a significant prognostic factor for OS (*p* = 0.136) (Table 2). Whereas, PFS was significantly different if complete cytoreduction was performed upfront in PCS or in ICS (13 months and 13.6 months respectively) when compared to 10.6 months for FCS (*p* = 0.019).

4. Only 214 patients were not eligible for surgery. Multivariate analysis showed that the decision to operate patients or not was influenced by FIGO stage IV versus stage III (OR 0.434 [CI 95% 0.311–0.606] *p* < 0.001), age > 75 years old versus age < 50 years old (OR 0.198 [IC95% 0.113–0.348] *p* < 0.001) and the period of treatment: after 2009 versus prior to 2000 (OR = 0.471 [IC95% 0.314–0.705] *p* < 0.001).

### 3.3. Evolution of Practices

The number of FIGO stage III-IV patients who underwent surgery decreased from 89.9% before 2000 to 77.4% after 2009 (*p* < 0.001). Since 1985, the rate of NAC increased parallel to the rate of complete surgery, from 37% before 2000 to 66.2% after 2009, in the total population study (Table 3).

Median OS improved during the four successive periods (*p* < 0.0001): median OS of the global study population was 33.1 months for patients managed during P1 and 57.8 months during P4 (*p* < 0.001). For patients who underwent complete cytoreduction, median OS was 47.7 months for P1 and 59.3 months for P4 (*p* = 0.015). In this group, median PFS was 10 months for P1 and 15 months for P4 (*p* = 0.002).

### 3.4. Multivariate Analysis

For the 1260 patients who underwent surgery, in multivariate analysis and after adjustment for histological type, time of surgery, FIGO stage at diagnosis, age, and residual disease after surgery, the following negative prognostic factors for OS were identified: FIGO stage, age, and complete resection (HR = 2.123, IC95% (1.816–2.481) *p* < 0.001 in case of residual disease).

**Figure 1:**
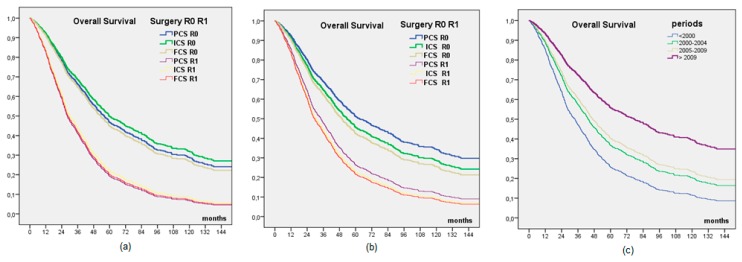
Multivariate analysis after adjustment for the prognostic factors; (**a**): overall survival depending on time of surgery and residual disease; (**b**): Cox model adjusted on periods; (**c**): overall survival depending on time periods.

Among patients with complete cytoreduction, after adjusting for the prognostic factors (Figure 1, Table 4), OS was no different for PCS, ICS, or FCS surgeries, but we observed a significant difference between FCS and PCS in an adjusted model on periods (HR = 1.278, CI 95% 1.027–1.590, *p* = 0.028), with a significant increase in OS from P1 to P4. PFS was significantly shorter for FCS (OR = 0.669, CI 95% 0.486–0.921, *p* = 0.014).

## 4. Discussion

Our retrospective analysis, evaluated the management of stage III–IV EOC in two comprehensive tertiary cancer centers. Our results concluded to a significant improvement in both OS and PFS associated with the increasing rate of complete cytoreductive surgery. OS was not statistically different between the different surgical strategies (PCS, ICS, and FCS), but PFS was shorter in cases of FCS.

### 4.1. Residual Disease after Surgery

During the last 30 years, 85.5% of our patients with EOC underwent surgery. A complete resection was achieved in 64.7% patients. The rate of complete cytoreduction (R0 surgeries) was higher when patients received NAC, it was 62.1% for PCS, 81.7% for ICS, and 78.1% for FCS. These results are consistent with those of a French multicentric study, in which the rate of R0 surgeries for 527 patients was 65% for PCS and 74% for ICS [23]. In a recent publication investigating the addition of HIPEC, van Driel et al. observed a similar result with a 67.7% complete cytoreduction rate for ICS [15].

During the study period, we witnessed an evolution of the goal of surgery that progressed from optimal cytoreduction to complete cytoreduction with no macroscopic residual disease at the end of surgery.

In 2002, Bristow et al. demonstrated in their meta-analysis [10] of 6885 patients that every 10% increase in maximal cytoreduction is associated with a 5.5% increase in median survival; maximal cytoreduction was one of the most powerful factors of survival among patients with stage III–IV EOC.

Other trials were in line with this conclusion: Chi et al. [11], showed that residual disease was the strongest prognostic factor; Winter et al. [24] showed in a retrospective study of 360 patients, that patients with residual disease of more than 5 cm had a diminished PFS and OS when compared with groups with less than 5 cm residual tumor.

With the goal of avoiding residual disease after surgery, Aletti et al. [9] showed that removal of diaphragmatic carcinomatosis lesions improved survival rate when compared with optimal resection without sub-diaphragmatic peritonectomy.

In our study, up to 90% of patients treated before 2000 underwent surgery but the complete resection rate did not exceed 50%. After 2009, 77% of patients underwent surgery and complete cytoreduction was achieved in 86%.

### 4.2. Type of Surgery

Over the study duration we witnessed an evolution in practices: prior to 2000, almost 70% of patients underwent PCS while after 2009 more than 50% of patients underwent surgery after NAC. Luycks et al. also described an increased use of NAC. In this comparable French study between 2003 and 2007, 36% of surgeries were PCS, 50% were ICS, and 13% were FCS; FCS was more frequently performed in our study [23].

Bristow et al. concluded in their meta-analysis in 2006, [25] that NAC, was associated with reduced OS when compared with primary cytoreduction. The negative relation between the number of NAC cycles and the survival of patients suggests that surgery should be performed as early as possible. The reported median OS for ICS patients in the Bristow et al. meta-analysis was 24.5 months versus 44.9 months in our study, with a complete cytoreduction rate of 81.7%. After literature review, six studies out of 22 defined the optimal residual disease size of <1 cm, others <2 cm, and PCS is currently considered as the gold standard strategy in all trials [17,18,26,27].

In 2010, a trial led by Vergote et al. [17] proved the non-inferiority of ICS when compared to PCS. Among 670 patients treated for EOC with FIGO stages IIIC or IV between 1998 and 2006, the median OS were 29 and 30 months, respectively, in PCS and ICS groups, PFS was 12 months in both groups. A complete resection was a strong and independent factor for predicting survival. The study concluded that NAC followed by ICS was non-inferior to PCS in terms of OS and PFS and was a valid therapeutic option for EOC with FIGO stages IIIC or IV. These results influenced our practices since it led to a decrease in the rate of PCS after the publication of the above mentioned trial.

But critics [28] have raised attention regarding the quality of cytoreduction in Vergote et al.’s study: for patients who underwent PCS complete resection was performed in 19.4% of cases and 22.2% had a residual tumor <1 cm. For ICS only 51.2% underwent complete resection (R0) and 47.2% had residual tumor. This criteria does not follow the current standards of quality for ovarian cytoreduction [28,29], knowing that complete cytoreduction is the major independent factor to improve survival.

In 2013, the EORTC trial results [26] suggested that patients presenting EOC stages IIIC or less had higher survival rates when treated with PCS, whilst patients with stage IV disease and large metastatic tumors had higher survival rates after NAC and ICS. For patients who did not meet these criteria, both treatment options led to comparable survival rates.

In 2015, the CHORUS phase III trial [18] randomized 552 patients for PCS or ICS. The results showed similar median OS of 22.6 and 24 months and similar PFS of 12 and 10.7 months for PCS and ICS, respectively. Less severe complications and rehospitalization were reported for ICS (*p* = 0.007). Quality of life was similar at 12-months follow-up. This trial also concluded to the non-inferiority of ICS vs. PCS. The main critique to this study was the low rate of complete resection in both, PCS (17%) or ICS (39%).

To our knowledge, data in literature is scarce concerning FCS although it represented 33% of the patients treated in our study since 2009.

In 2013, Da costa Miranda et al.’s study [20] retrospectively analyzed 82 patients who were initially considered not candidates for complete cytoreduction due to the extent of their disease. Patients had six to eight cycles of chemotherapy followed by FCS with no adjuvant chemotherapy. Median OS was 37 months (42 months in case of complete resection), which is comparable to our results, and PFS was 16 months with a 63% complete resection rate. After chemotherapy, 23% patients had complete response to chemotherapy, 57% presented a partial response, 5% had a stable disease and 12% had a progressive disease. The authors concluded to comparable survival rates between FCS and other surgical strategies which is in line with data in literature [10,17]. FCS was associated with fewer complications and better ease of surgery scheduling. Stoeckle et al. retrospectively [21] observed 118 patients who underwent FCS (late ICS, after eight cycles of chemotherapy), 81% had residual disease after surgery. Median OS was 42 months and PFS 17 months. Bowel resection was associated with a higher complication rate. They concluded that FCS offers equivalent survival and is beneficial for patients with stage IV EOC and for patients with rectal involvement. However, the absence of a control group makes both these studies questionable in terms of methodological approach.

In our study, 423 patients underwent FCS. We found a higher rate of complete resection after NAC but with lesser OS rate in multivariate analysis adjusted to different periods of time. PFS was shorter in case of FCS (HR = 0.669; IC95% 0.486–0.921, *p* = 0.014).

Recent publications suggest that the optimal management of patients presenting advanced EOC should include a multidisciplinary approach in order to define the best treatment strategy for each patient [30]. The aim is to have the highest rate of complete resection and to postpone the surgery if the patient is vulnerable or the extension of the disease does not allow complete resection.

### 4.3. Surgical Morbidity

A limitation of the current study is missing data concerning surgical morbidity. However, knowing that the two pillars for EOC management are surgery and chemotherapy combined, published data confirm that after complete cytoreduction, the delay in starting adjuvant chemotherapy is associated with shorter OS and PFS [31,32]. This was not established in case of macroscopic residual disease after surgery. In their trial, Eskander et al. [33] showed that post-operative 30 days readmission rate was 19.5% for patients who underwent surgery for EOC, and that delayed chemotherapy resulted in higher mortality rates: 41.1% vs. 25.1% *p* < 0.001.

Aletti et al.’s trial [30] aimed to identify EOC patients at highest risk for morbidity and mortality who underwent aggressive PCS followed by chemotherapy. FIGO stage IV, poor physical performance, poor nutritional status and advanced age were factors associated with high morbidity rates and limited survival benefit. However, in their trial, Shalowitz et al. emphasized that without treatment, survival for EOC patients is less than two months, and with chemotherapy alone, survival is less than one year [34]. This shows that patients not treated surgically have a poorer prognosis. Treatment should be planned for each patient taking into consideration clinical prognostic factors (age, performance, and nutritional status) and histo-pathological factors (FIGO stage, tumor’s histology), to identify the best surgical strategy, achieve the most complete cytoreduction, and decrease the delay of adjuvant chemotherapy. This allows tailoring the treatment for high risk patients; thus, improving EOC prognosis.

### 4.4. Limitations

Clinical and pathological features of our study population were similar to data published in literature [1,10]. We noticed an evolution in the regimens of systemic therapy over the years with a significant switch towards platinum and taxanes based regimens after the year 2000 (*p* < 0.001) and this is in line with the findings of the meta-analysis performed by Kyrgiou et al. [35].The surgical rate in our analysis [34], the complete resection rate and the NAC rate were similar to published data [23]. The evolution of therapeutic approaches over the study period (1985–2015) led to a certain heterogeneity in the treatments strategies but has also permitted the collection of a large and rich database. The retrospective aspect of the study induced missing data concerning the OS and PFS specific to each chemotherapy regimen and the amount of residual disease after surgery which reduces the power of our study, since this is a major prognostic factor in EOC management. Finally, additional adjuvant chemotherapy after FCS was not analyzed in this study, but this question will be one of the objectives of the ongoing CHRONO trial (NCT03579394).

### 4.5. Future Trials

Currently, two important questions remain unanswered concerning the surgical management for EOC:

When is the optimal timing for surgical cytoreduction if complete cytoreduction is deemed possible? Since the Vergote et al. [17] and CHORUS [18] trials, it is known that PCS and ICS are equivalent in terms of OS and PFS. Our analysis confirms this hypothesis. In addition, the TRUST trial (Trial of Radical Upfront Surgical Therapy in Advanced Ovarian Cancer) [36] aims to clarify the optimal timing of surgical therapy in patients managed for FIGO stage IIIB, IIIC, and resectable stage IV. To guarantee surgical quality, quality assurance criteria need to be respected: >50% complete resection rate in upfront surgery and ≥36 debulking-surgeries/year by participating centers.

When is the best timing for surgical cytoreduction when complete resection is deemed impossible at first? At present, surgery is performed after three to four cycles of chemotherapy, in ICS, or after six cycles in FCS.

Our results show that FCS does not increase the rate of complete cytoreduction and is associated with a shorter PFS. Multivariate analysis concluded that those patients undergoing FCS are the ones that present the worst prognosis with an independently shorter PFS. (HR = 0.669 IC95%; 0.486–0.921; *p* = 0.014). However, very few data exist concerning FCS in the literature; the CHRONO multicentric prospective randomized trial will, maybe, answer to the question of the interest of delayed ICS after six cycles of chemotherapy followed by two additional cycles after surgery.

## 5. Conclusions

Our results showed a significant improvement in OS and PFS associated with enhanced complete cytoreduction with the time. Between 1985 and 2015, 85.5% of patients with EOC underwent surgery, out of which 52% had PCS, 14% ICS, and 34% FCS. Since 2009 and after the results of the Vergote et al. [17] and CHORUS trials [18], which proved the role of NAC in helping achieving a more complete cytoreduction with no decrease in OS, neoadjuvant approach was more widely adopted.

For patients presenting an extensive disease even after three cycles of NAC, FCS might be the last resort option in spite of a shorter PFS. Few trials studied FCS and showed that it might be an interesting and safe alternative in cases of a very extensive disease.

Our study analyzed treatment strategies over a long period of time during which we witnessed an evolution of treatment concepts and chemotherapy modalities. Our results are in line with the recommendations of complete surgery and define FCS as an acceptable alternative when complete cytoreduction is not achievable previously.

Further studies are necessary to identify the optimum treatment strategy that offers the best survival with an optimal quality of life and the least morbidity.

HIPEC during ICS, classifying EOC based on the molecular profiles and the use of targeted therapy present new perspectives for the management of this aggressive disease. 

## Figures and Tables

**Table 1:** Comparison of patients depending on surgical strategy and residual disease. (a) Comparison of patients depending on surgical strategy. (b) Comparison of patients depending on residual disease at the end of surgery.

**(a) Comparison of patients depending on surgical strategy.**										
**Surgical Strategy**		**Total**	**Surgery**					**No Surgery**		
			**PCS**		**ICS**	**FCS**				**Chi 2**
		**Nb (%)**	Nb (%*)		Nb (%*)	Nb (%*)		**Nb (%)**		***p***
Number of patients		1474	659 (44.7)		178 (12.1)	423(28.7)		214 (14.5)		
Age	<50	271 (18.4)	153 (56.6)		25 (9.2)	72 (26.6)		21 (7.7)		<0.0001
	50–74	1028 (69.7)	438 (42.6)		144 (14)	314 (30.5)		132 (12.8)		
	> = 75	175 (11.9)	68 (38.9)		9 (5.1)	37 (21.1)		61 (34.9)		
FIGO Stage	III	1170 (79.4)	562 (48)		149 (12.7)	317 (27.1)		142(12.1)		<0.0001
	IV	304 (20.6)	97 (31.9)		29 (9.5)	106 (34.9)		72(23.7)		
Histological type	Serous	958 (65.2)	405 (42.3)		138 (14.4)	286 (29.9)		129(13.5)		<0.0001
	Mucinous	90 (6.1)	59 (65.6)		3 (3.3)	12 (13.3)		16 (17.8)		
	Endometrioid	105 (7.1)	68 (64.8)		7 (6.7)	24 (22.9)		6 (5.7)		
	Clear cells	49 (3.3)	28 (57.1)		2 (4.1)	15 (30.6)		4 (8.2)		
	other	124 (8.4)	32 (25.8)		20 (16.1)	44 (35.5)		28 (22.6)		
	undifferentiated	113 (7.7)	47 (41.6)		7 (6.2)	30 (26.5)		29 (25.7)		
	carcinosarcoma	20 (1.4)	12 (60)		1 (5)	5 (25)		2 (10)		
	Mixed	11 (0.7)	6 (54.5)		0 (0)	5 (45.5)		0 (0)		
CA 125**	<500	125 (40.5)	85 (48.3)		12 (28.6)	28 (30.8)		DM		0.004
	500–1000	64 (20.7)	32 (18.2)		10 (23.8)	22 (24.2)		DM		
	>1000	120 (38.8)	59 (33.5)		20 (47.6)	41 (45.1)		DM		
	DM	1165								
Chemotherapy protocol	Platinum + Taxanes	1076 (73.0)	391 (61.8)		161 (90.4)	355 (85.5)		169 (79.3)		<0.0001
	Other	363 (24.6)	242 (38.2)		17 (9.6)	60 (14.5)		44 (20.7)		
	DM	35 (2.4)								
Cancer Institute	1	685 (46.5)	189 (27.6)		128 (18.7)	271 (39.6)		97(14.2)		<0.0001
	2	789 (53.5)	470 (59.6)		50 (6.3)	152 (19.3)		117(14.8)		
**(b) Comparison of patients depending on residual disease at the end of surgery.**										
**Population’s Characteristics**				**Residual Disease**						
				**No**			**Yes**		**Chi 2**	
		**Missing data (*n* = 92, 7.3%)**		**Nb (%)**			**Nb (%)**		***p***	
Surgery	PCS	18 (2.7%)		398 (62.1)			243 (37.9)		<0.0001	
	ICS	25 (14%)		125 (81.7)			28 (18.3)			
	FCS	49 (11.6%)		292 (78.1)			82 (21.9)			
FIGO Stage	III			684 (71.2)			277 (28.8)		0.016	
	IV			131 (63.3)			76 (36.7)			
Histological type	Serous			566 (74.2)			197 (25.8)		<0.0001	
	Mucinous			30 (42.9)			40 (57.1)			
	Endometrioid			61 (62.2)			37 (37.8)			
	Clear cells			29 (72.5)			11 (27.5)			
	other			63 (73.3)			23 (27.5)			
	undifferentiated			47 (59.5)			32 (26.7)			
	carcinosarcoma			10 (55.6)			8 (44.4)			
	Mixed			6 (60)			4 (40)			
Periods	<2000			165 (49.7)			167 (50.3)		<0.0001	
	2000–2004			144 (64.6)			79 (35.4)			
	2005–2009			193 (77.5)			56 (22.5)			
	>2009			313 (86)			51 (14)			
Age	<50			166 (73.1)			61 (26.9)		0.167	
	50–74			579 (69.8)			251 (30.2)			
	> = 75			70 (63.1)			41 (36.9)			
CA 125	<500			83 (67.5)			40 (32.5)		0.368	
	500–1000			44 (69.8)			19 (30.2)			
	>1000			87 (75.7)			28 (24.3)			

%*: percent of total population (including cases without surgery); CA125**: percent under CA125 rate depending on surgical time; DM: missing data; Nb: number of patients. Primary Cytoreductive Surgery (PCS); Interval Cytoreductive Surgery (ICS); Final Cytoreductive Surgery (FCS); Chi-squared test (Chi 2); International Federation of Gynecology and Obstetrics (FIGO).

**Table 2:** Overall survival and Progression Free survival for patients with Complete Cytoreduction (R0).

Survival Outcome		Overall Survival			Progression Free Survival			
Characteristics		n*	Median of Survival (months)	*p*	n*	n** PFS < 12 m (%*)	n** PFS > 12m (%*)	*p*
R0		815	55.1		770			
Surgery	PCS	398	59.7	0.136	376	169 (45)	207 (55)	<0.001
	ICS	125	56.0		112	40 (36)	72 (64)	
	FCS	292	48.6		282	163 (58)	119 (42)	
Age	< 50	166	78.1	0.002	158	65 (41)	93 (59)	0.089
	50–74	579	53.6		543	269 (50)	274 (50)	
	>= 75	70	41.7		69	38 (55)	31 (45)	
FIGO	III	684	57.2	0.005	654	294 (45)	360 (55)	<0.001
	IV	131	44.9		116	78 (67)	38 (33)	
Histological type°	Serous	566	55.8	0.012	541	263 (49)	278 (51)	0.163
	Mucinous	30	67.1		28	13 (46)	15 (54)	
	Endometrioid	61	57.9		56	24 (43)	32 (57)	
	Clear cells	29	33.2		26	17 (65)	9 (35)	
	other	63	77.8		58	23 (40)	35 (60)	
	undifferentiated	47	51.8		44	20 (45)	24 (55)	
	carcinosarcoma	10	49.3		10	6 (60)	4 (40)	
	Mixed	6	9.6		4	4 (100)	0 (0)	
Periods	<2000	165	47.7	0.015	154	80 (52)	74 (48)	0.169
	2000–2004	144	54.3		135	54 (40)	81 (60)	
	2005–2009	193	59.0		189	91 (48)	98 (52)	
	>2009	313	59.3		292	147 (50)	145 (50)	
Cancer Institute	1	388	61.5	0.002	366	152 (42)	214 (58)	<0.001
	2	427	50.6		404	220 (54)	184 (46)	

n*: 815 patients with complete surgery, 770 patients with data concerning Progression-Free Survival (PFS). n**: number of patients with PFS < or > 12 months. °: for histological type: Overall Survival (OS) on 812 patients, PFS on 767 patients. % *: percent of patients with PFS < or > 12 months.

**Table 3:** Comparison of support based on the periods.

		Periods				
		<2000	2000–2004	2005–2009	>2009	
		Nb (%*)	Nb (%)	Nb (%)	Nb (%)	*p*
Surgery	Yes	401 (89.9)	240 (90.2)	253 (87.5)	366 (77.4)	<0.001
	No	45 (10.1)	26 (9.8)	36 (12.5)	107 (22.6)	
Strategy	PCS	311 (69.7)	131 (49.2)	98 (33.9)	119 (25.2)	<0.001
	ICS	22 (4.9)	39 (14.7)	27 (9.3)	90 (19.0)	
	FCS	68 (15.2)	70 (16.5)	128 (44.3)	157 (33.2)	
	No surgery	45 (10.1)	26 (9.8)	36 (12.5)	107 (22.6)	
Residual Disease	Yes	281 (63.0)	122 (45.9)	96 (33.2)	160 (33.8)	<0.001
	No	165 (37.0)	144 (54.1)	193 (66.8)	313 (66.2)	
Chemotherapy protocol	Platinum + Taxanes	94 (21.1)	233 (87.9)	282 (97.5)	467 (98.7)	<0.001
	Other	329 (73.7)	25 (9.4)	6 (2)	3 (0.1)	

*: percentage among total population, Nb: number of patients.

**Table 4:** Factors influencing Overall Survival (OS) and PFS among R0 patients. (a) Factors influencing overall survival among R0 patients. (b) Factors influencing progression free survival among R0 patients.

**(** **a) Factors influencing overall survival among R0 patients.**				
**Overall Survival**				
		**HR**	**IC95%**	***p***
Surgery	PCS	1		
	ICS	1.001	0.734–1.366	0.994
	FCS	1.123	0.906–1.393	0.289
FIGO Stage	III	1		
	IV	1.298	0.995–1.694	0.054
Age	< 50	1		
	50–74	1.355	1.061–1.729	0.015
	>= 75	1.951	1.318–2.888	0.001
Histological type	Serous	1		
	Mucinous	0.911	0.549–1.513	0.719
	Endometrioid	0.984	0.692–1.400	0.929
	Clear cells	1.668	1.055–2.638	0.029
	other	0.673	0.446–1.014	0.058
	undifferentiated	1.178	0.805–1.722	0.399
	carcinosarcoma	1.042	0.463–2.344	0.921
	Mixed	2.996	1.222–7.344	0.016
**(b) Factors influencing progression free survival among R0 patients.**				
**PFS**		**PFS <12 vs. >12 months**		
		**OR**	**IC95%**	***p***
Surgery	PCS	1		
	ICS	1.526	0.982–2.373	0.060
	FCS	0.669	0.486–0.921	0.014
FIGO Stage	III	1		
	IV	0.441	0.288–0.677	<0.001

**Figure A1:**
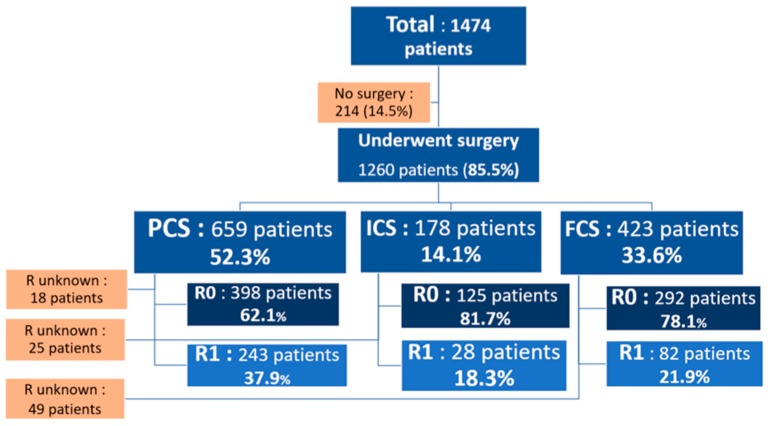
Flowchart. R0: complete resection of all visible disease, R1: remaining small volume of residual disease.
